# Indictor 4, promoting evidence informed community safety promotion

**Published:** 2019-08

**Authors:** Dale Hanson

**Affiliations:** ^*a*^Associate Professor, Chair. International Safe Community Certifying Center, Australia.

## Abstract

**Background:**

Designing a safety promotion programs that have significant impact on the wellbeing of your community is about finding creative solutions for the tension between visionary dreams and practical, evidence-informed solutions: • Nothing will be achieved unless you have the vision to imagine a better reality for ourselves, our family, our friends and our community • Nothing can be achieved unless you have the pragmatism and commitment to seek out achievable evidence informed effective solutions1. To achieve population level reductions in injury you need to GET REAL!

**Purpose:**

The ISCCC commissioned a guideline to assist communities addressing Indicator 4 - Programs that are based on available evidence - in their designation application.

**Methods:**

Review of the international literature on translation of research into practice.

**Results:**

Millions of lives could be saved if we just applied what is already known. This is the business of Indicator 4. Effective public health programs are the logical consequence of sound evidence. To implement an effective community-based safety promotion and injury prevention program three types of experts are required 1. Researchers (experts in what to do) 2. Politicians, administrators and practitioners (experts in how to do it) 3. Members of the target community (experts in what will work for them) four types of knowledge / evidence are required: 1. Evidence from a community’s own research and surveillance systems (Indicator 6) 2. Evidence about common types of injury and their causes 3. Evidence-based practice – evidence about what has been shown to work under research conditions 4. Practice based evidence – evidence about what has been shown to work in real world conditions.

**Conclusions:**

Accessing, interpreting and applying sound evidence is critical to the success of a safe community program.

**Keywords:**

Safe Communities, Designation, Evidence-based practice, Practice-based evidence, Knowledge Translation

